# Fallacies of Using the Win Ratio in Cardiovascular Trials: Challenges and Solutions

**DOI:** 10.1016/j.jacbts.2023.05.004

**Published:** 2023-06-26

**Authors:** Ezimamaka Ajufo, Aditi Nayak, Mandeep R. Mehra

**Affiliations:** Center for Advanced Heart Disease, Brigham and Women’s Hospital, Harvard Medical School, Boston, Massachusetts, USA

**Keywords:** cardiovascular trials, clinical significance, hierarchal endpoints, statistics, win odds, win ratio

## Abstract

The win ratio was introduced into cardiovascular trials as a potentially better way of analyzing composite endpoints to account for the hierarchy of clinical significance of their components and to facilitate the inclusion of recurrent events. The basic concept of the win ratio is to define a hierarchy of clinical importance within the components of the composite outcome, form all possible pairs by comparing every subject in the treatment group with every subject in the control group, and then evaluate each pair for the occurrence of the components of the composite outcome in descending order of importance, starting at the most important and progressing down the hierarchy if the outcome does not result in a win in either pair until pairs are tied for the outcome after exhaustion of all components. Although the win ratio offers a novel method of depiction of outcomes in clinical trials, its advantages may be counterbalanced by several fallacies (such as ignoring ties and weighting each hierarchal component equally) and challenges in appropriate clinical interpretation (establishing clinical meaningfulness of the observed effect size). From this perspective, we discuss these and other fallacies and provide a suggested framework to overcome such limitations to enhance utility of this statistical method across the clinical trial enterprise.

The efficacy of cardiovascular medicines and devices is commonly tested in clinical trials using composite endpoints that combine fatal and nonfatal events,^1^ and the U.S. Food and Drug Administration (FDA) recently released guidance to inform this well-established practice.^2^ Traditionally, the effect of an intervention on a composite endpoint is evaluated by comparing the time to occurrence of the first component of the composite event in the treatment or control arm of the study using the Cox model, which estimates event-free survival after adjusting for other important clarifying variables. The advantages of the proportional hazards or traditional approach are its simplicity and reproducibility. However, by taking only the first event into account, it is disproportionately influenced by nonfatal events (which typically tend to occur more frequently and before fatal events), under-represents fatal events, and ignores recurrent events (unless specific analytical methods are applied).^3^ A growing awareness of these limitations has inspired novel approaches to analyze clinical trials that are designed using composite endpoints. In 2012, the win ratio (WR) was reintroduced into cardiovascular trials as a potentially better way of analyzing composite endpoints to account for the hierarchy of clinical significance of their components and to facilitate inclusion of recurrent events. Pocock and colleagues described the WR method to estimate a treatment effect (CI and *p* value) based on the principle of generalized pairwise comparisons previously introduced by Finkelstein and Schoenfeld in 1999.^4^^,^^5^ The basic concept of the WR is to define a hierarchy of clinical importance within the components of the composite outcome, form all possible pairs by comparing every subject in the treatment group with every subject in the control group, and then evaluate each pair for the occurrence of the components of the composite outcome in descending order of importance, starting at the most important and progressing down the hierarchy if the outcome does not result in a win in either pair until pairs are tied for the outcome after exhaustion of all components (Figure 1). The WR is calculated as the total number of wins in the treatment group divided by losses in this group (or wins in the control arm). The growing acceptance of the WR had led to a widespread influx of trials incorporating it in either their primary or secondary analyses, largely because of its simplicity in performance and simplicity in interpretation of whether one treatment is a winner or not. It is of critical importance that the cardiovascular community familiarize itself with the concept of the WR, appreciate its interpretation, and, more important, its limitations. In this perspective, we outline major fallacies of the WR and, where possible, we propose solutions for use and interpretation.

**Figure 1:**
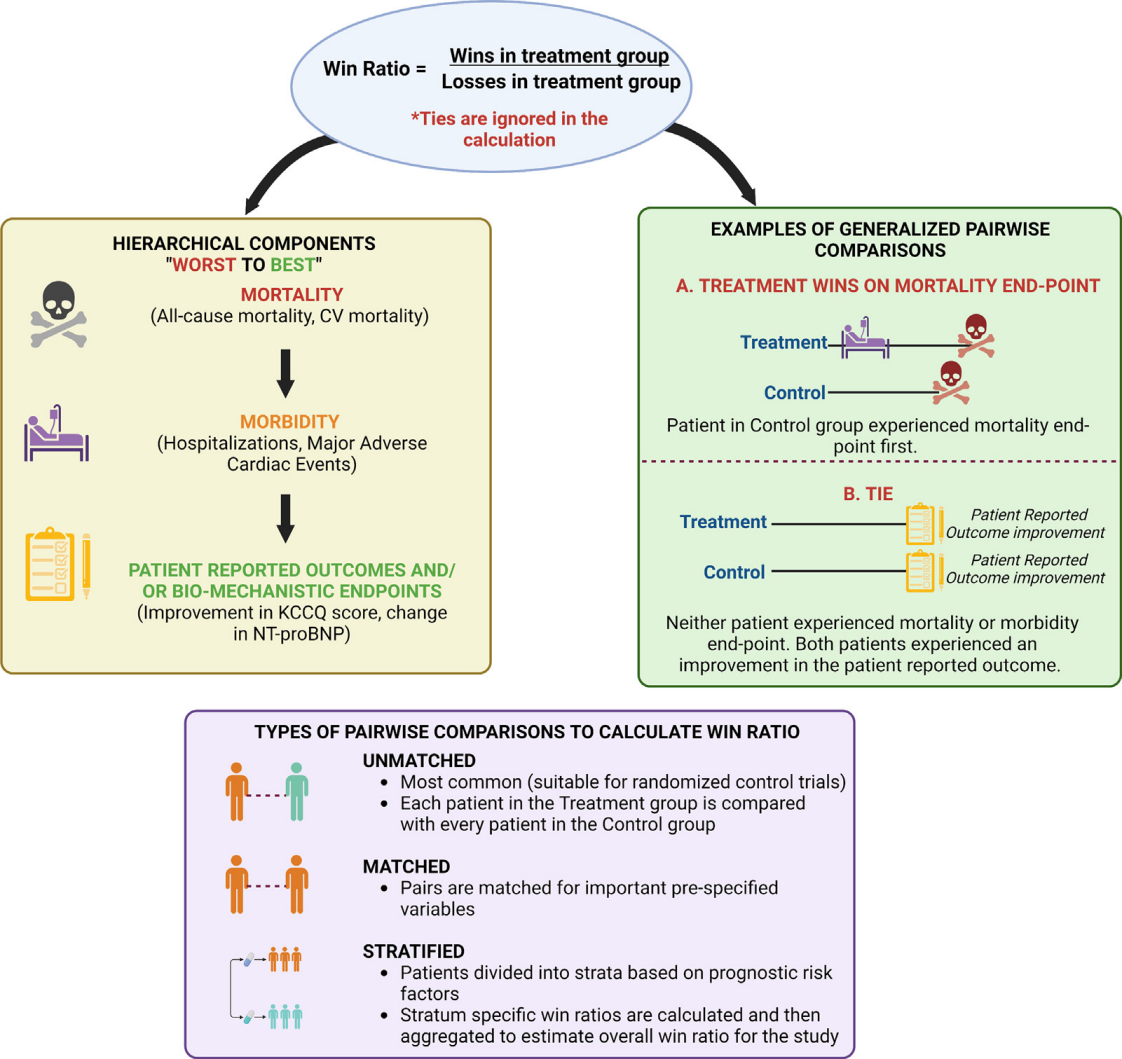
The Win Ratio Methodology Explained First, a hierarchy of clinical importance is defined within the components of the composite outcome (typically ordered as mortality endpoints > morbidity endpoints > patient-reported outcomes, and/or biomechanistic endpoints). Generalized pairwise comparisons are then performed, comparing each patient in the treatment group with every patient in the control group. Pairs are compared for the occurrence of the components of the composite outcome in descending order of importance, progressing down the hierarchy if the outcome does not result in a win in either pair until pairs are tied for the outcome after exhaustion of all components. The win ratio is calculated as the total number of wins in the treatment group divided by losses in this group. Importantly, ties are ignored. Modifications to the pairwise comparison method have been developed for studies that are nonrandomized or have a baseline imbalance in risk prognosticators between the treatment and control arms. These include matched pair analyses in which the pairs are matched for prespecified variables of importance, and stratified analyses in which patients are divided into strata based on their risk, strata-specific win ratios are calculated and aggregated for the overall win ratio. KCCQ = Kansas City Cardiomyopathy Questionnaire; NT-proBNP = N-terminal pro B-type natriuretic peptide.

## Fallacy 1*:* The Win Ratio can be Interpreted Like a Hazard Ratio to Depict Overall Treatment Effect

The hazard ratio (HR) calculated from the Cox proportional hazards model provides a ratio of the event rate in the treatment to the control arm at any given time during the study period. A HR of 0.75 denotes a 25% decrease in the event rate in the treatment group compared to the control group. By contrast, a WR of 1.25, which ignores pairs that are tied, simply denotes that, among the smaller subgroup of those able to be segregated into discrete wins or losses, the proportional win rate is 25% greater for a treatment under assessment. This strategy does not allow for an accurate calculation of the number needed to treat or harm, unlike what can be accomplished from examining the absolute risk reduction between groups using traditional statistical methods. In the ATTR-ACT (Tafamidis in Transthyretin Cardiomyopathy Clinical Trial), which examined the efficacy of tafamidis on the composite of all-cause mortality or cardiovascular-related hospitalizations in a population of individuals with transthyretin amyloid cardiomyopathy, the WR for the primary composite endpoint was 1.70, whereas the Cox model-based analyses suggested that the range of benefit was in the range of a 20% to 30% risk reduction.^6^ Thus, the magnitude of the overall effect cannot be interpreted easily simply from the WR, and translation of the magnitude of the population effect is not possible without examining the entirety of the population studied, inclusive of pairs of individuals that are tied in outcome. Furthermore, unlike a time-dependent analysis, a WR cannot convey if an effect occurred early or late during treatment, nor does it denote durability of the effect or its magnitude, such as one may calculate from differences among event-free survival curves over a well-defined follow-up period (eg, to calculate median prolongation in survival).^7^

## Fallacy 2: A Win Mostly Suggests a Clinically Meaningful Benefit

Historically, a small number of clinically meaningful composite endpoints have been used in the cardiovascular literature. There is familiarity with these endpoints, their advantages, and limitations and, importantly, there is scope for performing at least informal comparisons across trials that examine the same composite. By contrast, there is considerable variability in the composition of novel hierarchical endpoints analyzed with the WR. It is important to realize that, although hierarchically ordered, each endpoint component of the WR is weighted the same. Therefore, a significant WR could be entirely driven by its least clinically meaningful endpoint component. As an example, in the PARAGLIDE-HF (Prospective comparison of ARNI with ARB Given following stabiLization In DEcompensated HFpEF) trial, which compared sacubitril-valsartan with valsartan in patients with a left ventricular ejection fraction of >40% after a recent worsening heart failure (HF) event, a prespecified secondary outcome was a hierarchical composite outcome of, in descending order of significance, cardiovascular death, HF hospitalizations, urgent HF visits, and time averaged change in N-terminal pro B-type natriuretic peptide.^8^ With respect to this outcome in a subgroup of patients with a left ventricular ejection fraction of >40% and <60%, they reported a win in favor of the treatment group with a WR of 1.46 (95% CI: 1.09-1.95). However, a more traditional analysis of clinically meaningful endpoints reported a rate ratio for the recurrent composite cardiovascular endpoint of 0.61 (95% CI: 0.37-1.01), which did not reach significance. Also, there was a 50% greater incidence of symptomatic hypotension in the group treated with sacubitril-valsartan. This finding suggests that the win may have been driven predominantly by the least significant endpoint of the time averaged change in N-terminal pro B-type natriuretic peptide, which should not be weighted clinically the same as the cardiovascular endpoints of death, hospitalizations, or episodes of clinical worsening.^9^ In this scenario, the favorable WR provides an illusion of a high degree of clinical meaningfulness. In yet another situation, the overall WR may not accurately reflect outcomes if inconsistent effects of a treatment occur on various components of the composite outcome. Take as an example a high-risk treatment (as in immunosuppression trials or cancer chemotherapy) that may prolong life modestly, but at the cost of a greater proportion of nonfatal events over time. In such a bidirectional outcome of hierarchal events, the wins from deaths would be neutralized by the losses from nonfatal events leading to an ultimate neutral finding on the aggregate WR. In another distinct situation, a win or loss based on time-related differences to establish wins or losses can cause additional confusion. As an example, a treatment could win on death (or a hospitalization) if the control group has a similar number of events, but if they beat the controls by occurring a day later. Such an outcome may not be particularly clinically meaningful or substantially cost effective. Therefore, in trials where a treatment may delay the occurrence of an adverse outcome, it is important to report estimates of time-dependent outcomes such as those described by a traditional event free survival analysis of the composite endpoint and its components.

## Fallacy 3: Patient-Reported Outcomes can be Better Assessed Using a WR Analysis

An attractive feature of the WR is that it provides increased flexibility in the composition of the endpoint in allowing the use of different types of variables, including patient-reported outcomes in combination with traditional events. This factor has been touted as an advantage of providing aggregate meaningfulness, which on its surface is rational.^10^ One major challenge in using such end points relates to the study design, with respect to whether blinding is imposed in the trial design to overcome a placebo effect. The randomized TRILUMINATE (Trial to Evaluate Cardiovascular Outcomes in Patients Treated with the Tricuspid Valve Repair System) trial, which evaluated the impact of transcatheter edge-to-edge repair vs medical therapy for symptomatic severe tricuspid regurgitation used a primary composite endpoint that included death, HF hospitalization and improvement in quality of life measured with the Kansas City Cardiomyopathy Questionnaire (a >15-point improvement).^11^ The trial met its primary endpoint with a WR of 1.48 (95% CI: 1.06-2.13); however, a component review indicated that this was not driven by an effect on all-cause mortality or HF hospitalization but instead, was entirely attributable to a large effect on the Kansas City Cardiomyopathy Questionnaire quality-of-life metric.^11^ Although this is a rather meaningful effect in terms of the patient experience, the device-based study was open label and patients who received the tricuspid repair device were aware of their allocation as well as the outcome of the intervention. Surprisingly, no differences in diuretic doses used over time or in functional parameters (6-meter walk distance) were evident in the trial suggesting that the large patient-reported outcome measure benefit may have had a strong placebo component. Yet, this trial demonstrated the feasibility, safety, and durability of the intervention, which is a clinical success, recognizing that such patients tend to be poor candidates for surgical valve repair. We recommend that the meaningfulness of such composite endpoints must be placed into context by evidence of other physiologically supported beneficial variables that are unaffected by the placebo effect (eg, changes in natriuretic peptides) and reporting must provide component related WR analyses of all endpoints, to allow a better understanding of the overall treatment effect. Ideally, trials that include patient-reported outcomes in the primary endpoint using the WR should be subject to use of a placebo (in case of drugs) or a sham (in case of a device) control group with adequate blinding during endpoint evaluation.

## Fallacy 4: The Risk Profiles of Each Analyzed Pair are Well-Balanced

A prerequisite for the calculation of the WR is the formation of treatment and control group subject pairs. This can be done in several ways. The most used approach is the unmatched pairs approach. Here, all participants in the treatment group are compared with all participants in the control group (total number of pairs = total number of individuals in the treatment group × total number of individuals in the control group). The drawback of this approach is that a baseline imbalance in risk within an analyzed pair may bias the outcome (eg, a 40-year-old woman compared with a 70-year-old man with HF). To address this issue, a matched pairs approach has been proposed with the goal of forming pairs based on shared baseline risk.^10^ However, it is difficult to define all adjustment variables prospectively that require adjustment and, therefore, this technique is not often used. Another strategy is to use a stratified WR approach. The stratified approach involves identifying major risk factors for the outcome in advance and dividing participants into strata based on these risk factors before performing pairwise comparisons between each stratum.^10^ The stratum specific win proportions are then combined across the strata to estimate the overall WR for the study. The stratified WR was used in the ATTR-ACT trial where individuals were stratified according to TTR status (variant or wild-type) and baseline NYHA functional class.^6^ In a randomized controlled trial, baseline risk is generally balanced between the study arms and, therefore, an unmatched WR is acceptable. For nonrandomized trials or randomized trials with imbalanced baseline risk of major prognostic variables (eg, mutations in sarcomeric proteins in a hypertrophic cardiomyopathy trial, ejection fraction in a HF trial), a stratified WR approach should ideally be undertaken.

## Fallacy 5: “Ties” Among Treatment and Control Groups can be Ignored without Consequence

Perhaps one of the greatest issues with the WR is the fact that, in calculating the WR, when a tie occurs (neither a win nor a loss), it is largely placed aside and ignored. Cardiovascular trials that enroll many patients are typified by a greater frequency of ties that, when ignored, result in an overestimation of the treatment effect in a WR calculation. The WR is most accurate in estimating an overall effect when there are few or no ties (as one would encounter in calculating the outcomes of a stock market index over time, where ties are infrequent, and highs or lows dominate). One proposed solution for this is to calculate a win odds (WO). In the WO, one-half of all ties are added to the wins for the treatment arm and control arms, respectively.^12^^,^^13^ In the TRILUMINATE trial, there were 1,1348 wins (37% of the pairs) in the treatment group, 7,643 losses (25% of the pairs), and 11,634 ties (40% of the pairs).^11^ The overall WR of 1.48 would be discounted to much a lower value calculated ratio of a WO of 1.28 once ties are accounted for in the analysis. Without calculating the contribution of ties, the magnitude of the effect size is markedly overestimated. Similar scenarios are provided for reference in Table 1. In a situation where ties are minimal, the WR will approximate the WO; however, in most cases the WO should be preferred over the WR. In the event of a significant amount of censoring before the end of the study, there is no clear consensus on whether the WO or WR is superior. When a patient is lost to follow-up, they are censored from the study. Because each pair can only be compared for their shared study period, if an event occurred in the patient that remains after the other drops out, or an event occurs in the subject that drops out after they leave the study, this cannot be adequately accounted. This type of censoring makes the assessment of a win, loss, or tie unreliable. Some methods to adjust the win statistic for censoring have been proposed, but require further evaluation.^14^ If many dropouts are encountered in a WR analysis, a sensitivity analysis that adjusts for censoring should be mandated. The second major consequence of ignoring ties is that the WR only reflects nonidentical outcomes between the groups and can be misleading about the similarity of the distribution of outcomes in the study arms. This finding is particularly relevant in noninferiority trials, because ties might reflect comparable treatment effect and this would not be captured in the WR. For noninferiority trials, the WO should be the preferred analysis approach or the use of traditional models.

**Table 1:** A Comparison of Win Ratio and Win Odds in Clinical Trials

TRILUMINATE Impact of Transcatheter Edge-to-Edge Repair for Symptomatic Severe Tricuspid Regurgitation^a^			PARADISE-MI Sacubitril-Valsartan vs. Ramipril in Acute MI (Post-Hoc Analysis Including Events That Did Not Meet CEC Study Definitions)^b^			PARAGLIDE-HF Angiotensin-Neprilysin Inhibition in Patients With Mildly Reduced or Preserved Ejection Fraction and Worsening Heart Failure^c^		
Hierarchical composite outcome	Wins	Losses	Hierarchical composite outcome	Wins	Losses	Hierarchical composite outcome	Wins	Losses
Events								
All-cause mortality or TV surgery	2,884	2,644	CV death (CEC confirmed)	459,367	406,389	Time to CV death	2,172	1,520
HF hospitalization	1,948	2,871	CV death (CEC unconfirmed)	11,843	5,418	Recurrent events of HF hospitalization	7,492	6,840
Improvement over baseline of ≥15 points in KCCQ score	6,516	2,128	HF hospitalization (CEC confirmed)	359,680	339,064	Recurrent urgent HF visits	1,357	814
			HF hospitalization (CEC unconfirmed)	180,879	163,511	Change in NT-proBNP	15,147	12,649
			Outpatient symptomatic HF (CEC confirmed)	72,590	63,692			
			Outpatient symptomatic HF (CEC unconfirmed)	181,408	101,428			
	11,348	7,643		1,265,767	1,079,502		26,168	21,823
	11,634 ties			5,666,461 ties			17,427 ties	
Win ratio: 1.48 (95% CI: 1.06-2.13), *P* = 0.02			Win ratio: 1.17 (95% CI: 1.03-1.33), *P* = 0.02			Win ratio: 1.19 (95% CI: 0.93-1.52), *P* = 0.16		
Win odds: 1.28			Win odds: 1.05			Win odds: 1.14		

CEC = Clinical Events Committee; CV = cardiovascular; HF = heart failure; Kansas City Cardiomyopathy Questionnaire; NT-proBNP = N-terminal pro B-type natriuretic peptide; TV = tricuspid valve.

a Sorajja P, Whisenant B, Hamid N, et al; TRILUMINATE Pivotal Investigators. Transcatheter Repair for Patients with Tricuspid Regurgitation. *N Engl J Med*. 2023 Mar 4. Doi: 10.1056/NEJMoa2300525.

b Berwanger O, Pfeffer M, Claggett B, et al. Sacubitril/valsartan versus ramipril for patients with acute myocardial infarction: win-ratio analysis of the PARADISE-MI trial. *Eur J Heart Fail*. 2022;24(10):1918-1927.

c Mentz RJ, Ward JH, Hernandez AF, et al. Angiotensin-neprilysin inhibition in patients with mildly reduced or preserved ejection fraction and worsening heart failure. *J Am Coll Cardiol*. 2023. doi:10.1016/j.jacc.2023.04.019

## Clinical Interpretation and Suggested Solutions to Overcome Fallacies in Using The WR

**Figure 2:**
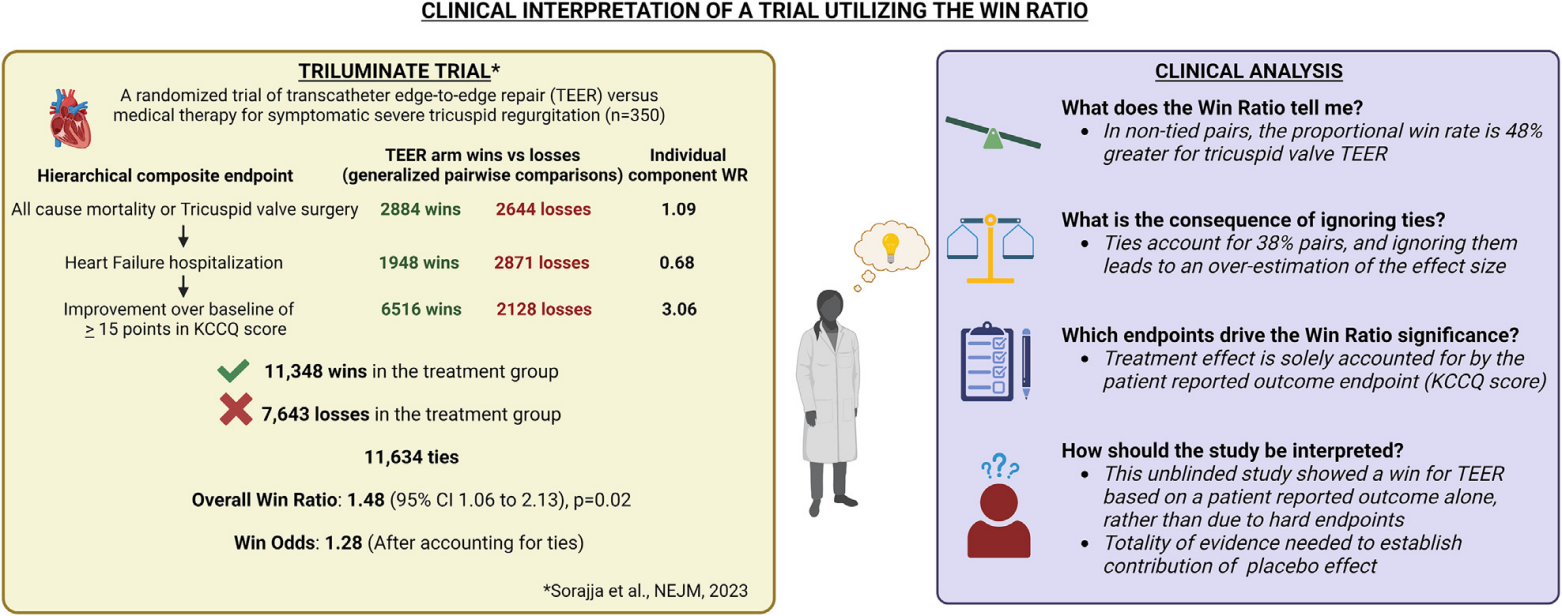
Critical Evaluation of a Clinical Trial Using the Win Ratio In this figure, we provide a framework for interpretation of clinical trials that use the win ratio using a representative clinical trial as an illustrative example. The trial was a prospective randomized trial of tricuspid valve transcatheter edge-to-edge repair (TEER) for patients with severe tricuspid regurgitation, without a sham-controlled arm (TRILUMINATE). The primary endpoint was a hierarchical composite (worst to best) of all-cause mortality or tricuspid valve surgery, hospitalization for heart failure or an improvement in Kansas City Cardiomyopathy Questionnaire (KCCQ) score of ≥15 points analyzed using the win ratio methodology. Generalized pairwise comparisons (comparing each patient in the TEER group with every patient in the control group) demonstrated an overall win on the primary endpoint for the TEER group (win ratio, 1.48; 95% CI: 1.06-2.13; *P* = 0.02). However, a clinical analysis demonstrates that: 1) ties accounted for 38% of the paired comparisons, and ignoring these ties leads to an overestimation of the effect size in this study; 2) the reported treatment effect is solely accounted for by the patient reported outcome endpoint, supported by the individual component win ratio calculations; and 3) the unblinded study design could have influenced the patient reported outcome endpoint. In such cases, we recommend examining the totality of evidence presented in the study, including other supportive surrogates and/or biomechanistic markers. Notably, in the TRILUMINATE study, there was no difference in 6-minute walk distance or in diuretic dosing between groups between the TEER and control groups at 1 year, suggesting a strong possibility that the patient reported measure may have been prone to a placebo effect.

The demonstration that an intervention has achieved its intended effects is an important legal and ethical standard that must be achieved before an intervention is introduced into general clinical practice. The statistical method by which this efficacy is established must undergo the highest level of scrutiny. We have described 5 fallacies of using the WR that highlight the complexity within this seemingly simple analytical method. As use of the WR becomes more common in cardiovascular trials, to complement the FDA’s recent guidance on the analysis of composite endpoints, we believe that the time has come to convene key stakeholders to outline consensus guidance on how (and when) the WR should be used in the analysis and interpretation of cardiovascular trials. We offer several suggestions for the optimal use and clinical interpretation of the WR within cardiovascular trials. A clinician should not stop interpretation at the WR statistic outcome alone. They must seek to understand the overall treatment effect (by accounting for ties and dropouts) and to have sufficient information from the analyses to ascertain a number needed to treat or harm. Thus, an assessment of aggregate clinical benefit is necessary to translate information from trials that demonstrate wins using the WR to not only appreciate the magnitude of effect, but temporal relationships of outcomes, their durability, and finally the overall value (or cost effectiveness). Similarly, what drives the overall win must be clearly depicted and whether a placebo effect may exist (Figure 2).

We also offer some important solutions to overcome the inherent challenges described in the various fallacies of using the WR or when to avoid its use. First, we recommend the traditional frequentist statistical methods approach over the simplistic WR in select scenarios. For instance, if the treatment effect is likely to be limited to nonfatal outcomes and a minimal treatment effect on mortality is anticipated, enrolling a population with an otherwise high mortality rate (from competing risk) could result in a significant loss of statistical power for the WR (by convention, hierarchical endpoints typically include mortality). If significant dropout is anticipated (as with a drug that has a high adverse effect profile or a clinical scenario with multiple competing outcomes), the introduction of bias with censoring before study completion creates a greater risk in WR assessment. By contrast, the proportional hazards model includes a well-established method for dealing with such participants or use of a Fine-Gray competing risk model analysis should be used.^15^ A more traditional analysis approach should also be preferred over the WR, if the WR would not be expected to make much of a difference to the analytic outcome. As an example, when risk for recurrent events is low, little information is lost by picking the time to a first event methodology.^16^ Importantly, to ensure that worthy endpoints are constructed for use in trials, we recommend the development of consensus standards of hierarchical composite endpoints used when a WR is considered a primary analysis. This should be based on the input of stakeholders including patients, and clinical trialists, along with regulatory and reimbursement agencies such as the FDA and the Centers for Medicare and Medicaid Services. Such a consensus should define the components to be included, their measurement standard, ordering, and, for continuous variables, how they should be compared in terms of clinical meaningfulness and with attention to time dependency. We also recommend standards for reporting and interpreting win statistics (Central Illustration). If the WR is used, the WO should be presented as well with its corresponding statistics. The presentation of these summary statistics should be complemented by win statistics (wins, losses, ties) for the composite outcome and each of the components (wins, losses) of the hierarchal composite endpoint. In the final interpretation of the WR and WO, the drivers of the outcome should be acknowledged explicitly and, unless there is consistency of wins across the components and in the case of measures prone to placebo effects, clinical meaningfulness must not be concluded unless the totality of evidence, including physiological and biological outcomes, support an overall clinical effect. When a therapy wins or loses based on a time-based component, the durability of the effect size must be reported separate from the WR, and a value-based analysis should ideally be included in pivotal trials.

**Central Illustration:**
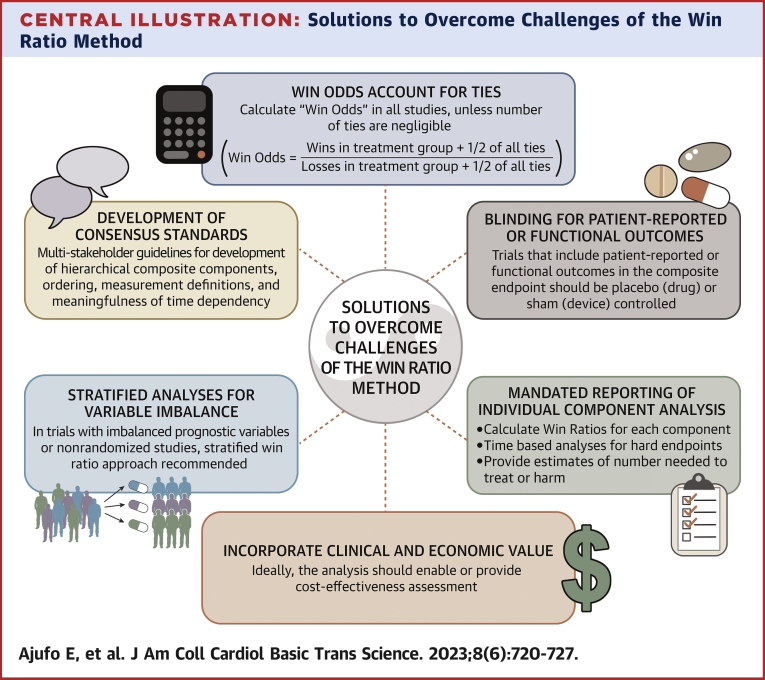
Solutions to Overcome Challenges of the Win Ratio Method We recommend several solutions to address the inherent fallacies of the win ratio statistical method: 1. Win Odds (that accounts for ties) should be reported for all studies that report the win ratio, unless the number of ties are negligible; 2. Trials that include patient reported or functional outcomes in the composite endpoint should be placebo or sham controlled to avoid biasing the overall results; 3. Individual end-point win ratios, time based analyses for hard end-points, and number needed to treat and harm should be clearly reported to determine magnitude of benefit; 4. The analyses should be designed to allow for conduct of cost-effectiveness analyses; 5. A stratified win ratio approach should be undertaken in trials with imbalanced baseline risk of major risk factors; 6. Multi-stake holder consensus standards (including patients, clinical trialists and governmental regulatory and reimbursement agencies) should be developed for design of hierarchical composite end-points to incorporate meaningfulness and reporting value.

In summary, although the WR offers a novel method of depiction of outcomes in clinical trials, its advantages may be counterbalanced by several potential fallacies in its conduct and clinical interpretation for which we have provided a framework to overcome such limitations and enhance the value of such utility across the clinical trial enterprise.

## Funding Support and Author Disclosures

Dr Mehra has received travel support and consulting fees, paid to Brigham and Women’s Hospital, from Abbott; fees for serving on a steering committee from Medtronic and Janssen (Johnson & Johnson); fees for serving on a data and safety monitoring board from Mesoblast; consulting fees from Natera, Paragonix, Moderna, the Baim Institute of Clinical Research, and Broadview Ventures; and fees for serving as a scientific board member from NuPulseCV, Leviticus, Transmedics and FineHeart. All other authors have reported that they have no relationships relevant to the contents of this paper to disclose.
